# Effects of BPA in Snails

**DOI:** 10.1289/ehp.114-1480521

**Published:** 2006-06

**Authors:** Daniel R. Dietrich, Evelyn O’Brien, Sebastian Hoffmann, Patrique Balaguer, Jean-Claude Nicolas, Willem Seinen, Michael Depledge

**Affiliations:** Human and Environmental Toxicology, University of Konstanz, Jacob-Burckhardstrasse, Germany, E-mail: daniel.dietrich@uni-konstanz.de; European Commission, Joint Research Centre, Institute of Health and Consumer Protection, Ispra, Italy; INSERM, Endocrinologie Moleculaire et Cellulaire, des Cancers, Montpellier, France; Institute for Risk Assessment Sciences, Utrecht, the Netherlands; Plymouth Marine Laboratories Plymouth, United Kingdom

It is an ethical requirement that new findings be presented in light of and in conjunction with a balanced evaluation of the current knowledge and published literature. We believe that Oehlmann et al. (2006) violated this general principle in several ways. For example, the authors inferred that prosobranch snails have a functional estrogen receptor and therefore a much higher sensitivity to estrogens and endocrine-disrupting compounds (EDCs) than other species previously reported in the literature. We found several other problems in their article:

First, Oehlmann et al. (2006) did not reveal the source of the animals used in their study, thus prohibiting independent repetition of the experiments by others.

Second, the authors stated that male and female *Marisa cornuarietis* cannot be distinguished morphologically without killing the animals. Therefore, the lack of data on the sex distribution of the animals sampled at each time-point leads us to question the stability of the experimental conditions with regard to sex ratios and thus reproductive conditions. Furthermore, the rapidly changing snail density, and hence the sex distribution at each sampling time point, certainly influenced the remaining animals with respect to mortality and fecundity.

Third, the experimental design and the lack of replication (Experiment 1) did not allow for sound statistical analysis; the statistical methods used were inappropriate, making correct interpretation impossible. Of most concern to us was the analysis of data by analysis of covariance (ANCOVA), mainly because the ANCOVA-inherent assumption of independency of the dependent variable (i.e., total number of eggs) is violated. Thus, small differences among aquaria (treatment groups) might have been propagated over time, resulting in the impression of large differences.

Fourth, we believe that carrying out receptor binding experiments only in duplicate and without Scatchard analysis is questionable per se. The number of concentrations tested was extremely limited and consequently cannot allow accurate description of binding curves. Oehlmann et al. (2006) provided no information regarding the assessment of unspecific binding and the reported IC_50_ values (concentration causing 50% inhibition) are approximately three orders of magnitude higher than what would be expected if this were a real sex-steroid receptor interaction. Because tamoxifen did not elicit a typical and highly specific receptor binding curve (Oehlmann et al. 2006, Figure 3), we question the use of tamoxifen as an “antiestrogen” in this *in vivo* study.

Finally, the data in Figure 1B (Oehlmann et al. 2006) were published earlier by Schulte-Oehlmann et al. (2001), yet the originally published data did not incorporate 17α-ethinylestradiol (EE_2_) as positive control. Moreover, the EE_2_ curve in Figure 1B appears identical to the one on slide 14 from a slide presentation available on Oehlmanns’ website (Schulte-Oehlmann et al. 2006).

The use of a positive control is commendable when the mode of action is known [National Toxicology Program (NTP) 2001]; however, as in the study of Oehlmann et al. (2006), the lack of such knowledge precludes the inclusion of a positive control as proof-of-principle. Slide 14 (Schulte-Oehlmann et al. 2006) demonstrates that EE_2_ does not have a monotonic mode of activity in *M. cornuarietis*, but rather appears to stimulate egg laying at 10–25 ng EE_2_/L, inhibit egg laying at 50 ng EE_2_/L and has no effect at 1 and 100 ng EE_2_/L. On the basis of *in vitro* and *in vivo* effects reported by Oehlmann et al. (2006), we question the presence of any estrogen receptor–like interaction. In view of the NTP (2001) definitions and use of controls, the use of EE_2_ as a “positive” control, with its nonmonotonic and nonhormetic dose–response curve in comparison with BPA (which has a presumably monotonic response curve), as well as the use of an antiestrogen (tamoxifen), is inappropriate.

In conclusion, the data presented by Oehlmann et. al. (2006) are unconvincing. Flaws in the experimental design, data presentation, and interpretation as well as statistical analyses render their findings untenable. Furthermore, the “Introduction” and “Discussion” of their article was written in a way that could be considered highly imbalanced and indeed alarmist. The highly selective inclusion/omission and discussion of previously published research that contradicts the authors’ opinion (e.g., Pickford et al. 2003) is particularly disturbing. It is our opinion that our evaluation of the Oehlmann et al. work serves as a useful reminder to scientists that we must constantly strive to formulate clear hypotheses, use sound experimental designs, employ appropriate statistics, and draw conclusions that are supported by the available data and that reflect a balanced assessment of the scientific literature to avoid jumping to erroneous conclusions.

## References

[b1-ehp0114-a00340] NTP. 2001. National Toxicology Program’s Report of the Endocrine Disruptors Low Dose Peer Review. Research Triangle Park, NC:National Toxicology Program. Available: http://ntp-server.niehs.nih.gov/ntp/htdocs/lia-son/LowDosePeerFinalRpt.pdf [accessed 14 April 2006].

[b2-ehp0114-a00340] Oehlmann J, Schulte-Oehlmann U, Bachmann J, Oetken M, Lutz I, Kloas W (2006). Bisphenol A induces super-feminization in the ramshorn snail *Marisa cornuarietis* (Gastropoda: Prosobranchia) at environmentally relevant concentrations. Environ Health Perspect.

[b3-ehp0114-a00340] Pickford DB, Hetheridge MJ, Caunter JE, Hall AT, Hutchinson TH (2003). Assessing chronic toxicity of bisphenol A to larvae of the African clawed frog (*Xenopus laevis*) in a flow-through exposure system. Chemosphere.

[b4-ehp0114-a00340] Schulte-Oehlmann U, Tillmann M, Casey D, Duft M, Markert B, Oehlmann J (2001). Öestrogenartige Wirkungen von Bisphenol A auf Vorderkiemenschnecken (Mollusca: Gastropoda: Prosobranchia). UWSF Z Umweltchem Ökotoxikol.

[b5-ehp0114-a00340] Schulte-Oehlmann J Nentwig G Oetken M Bachmann J Oehlmann J 2006. Effekte von ausgewählten Arzneimittel-wirkstoffen auf aquatische Wirbellose. Available: http://www.bio.uni-frankfurt.de/ee/ecotox/_files/teaching/hauptstudium/ecotox6.pdf [accessed 18 April 2006].

